# Evaluative Processing of Food Images: A Conditional Role for Viewing in Preference Formation

**DOI:** 10.3389/fpsyg.2018.00936

**Published:** 2018-06-11

**Authors:** Alexandra Wolf, Kajornvut Ounjai, Muneyoshi Takahashi, Shunsuke Kobayashi, Tetsuya Matsuda, Johan Lauwereyns

**Affiliations:** ^1^Graduate School of Systems Life Sciences, Kyushu University, Fukuoka, Japan; ^2^Brain Science Institute, Tamagawa University, Tokyo, Japan; ^3^Department of Neurology, Fukushima Medical University, Fukushima, Japan; ^4^Faculty of Arts and Science, Kyushu University, Fukuoka, Japan

**Keywords:** gaze duration, viewing time, self-paced versus time-controlled, non-exclusive versus exclusive, evaluative processing, naturalistic food images

## Abstract

Previous research suggested a role of gaze in preference formation, not merely as an expression of preference, but also as a causal influence. According to the gaze cascade hypothesis, the longer subjects look at an item, the more likely they are to develop a preference for it. However, to date the connection between viewing and liking has been investigated predominately with self-paced viewing conditions in which the subjects were required to select certain items from simultaneously presented stimuli on the basis of perceived visual attractiveness. Such conditions might promote a default, but non-mandatory connection between viewing and liking. To explore whether the connection is separable, we examined the evaluative processing of single naturalistic food images in a 2 × 2 design, conducted completely within subjects, in which we varied both the type of exposure (self-paced versus time-controlled) and the type of evaluation (non-exclusive versus exclusive). In the self-paced exclusive evaluation, longer viewing was associated with a higher likelihood of a positive evaluation. However, in the self-paced non-exclusive evaluation, the trend reversed such that longer viewing durations were associated with lesser ratings. Furthermore, in the time-controlled tasks, both with non-exclusive and exclusive evaluation, there was no significant relationship between the viewing duration and the evaluation. The overall pattern of results was consistent for viewing times measured in terms of exposure duration (i.e., the duration of stimulus presentation on the screen) and in terms of actual gaze duration (i.e., the amount of time the subject effectively gazed at the stimulus on the screen). The data indicated that viewing does not intrinsically lead to a higher evaluation when evaluating single food images; instead, the relationship between viewing duration and evaluation depends on the type of task. We suggest that self-determination of exposure duration may be a prerequisite for any influence from viewing time on evaluative processing, regardless of whether the influence is facilitative. Moreover, the purported facilitative link between viewing and liking appears to be limited to exclusive evaluation, when only a restricted number of items can be included in a chosen set.

## Introduction

Tracking the gaze provides a moment-by-moment assessment of thought processes in a wide variety of contexts (Shepherd et al., 1986; Liversedge and Findlay, 2000; Theeuwes et al., 2009; Tatler et al., 2010; Bulling et al., 2011). Visual behavior is tightly linked to learning (Heisz and Shore, 2008), associative memory (Hannula and Ranganath, 2009), saliency determination (Henderson, 2003), judgment tasks concerning products (Pärnamets et al., 2016), decision-making under uncertainty (Zommara et al., 2018), and moral dilemma problem solving (Pärnamets et al., 2015). Particularly gaze duration, or viewing time, appears to be a useful index of the extent of information processing (see Lauwereyns, 2012, for a comprehensive review). Here, we examine the role of viewing time in evaluative processing with respect to naturalistic food images.

The dominant hypothesis in the literature on evaluative processing is that viewing leads to increased evaluation. As an early reference in this vein, we note the classic work by Zajonc (1968) on attitudinal effects of mere exposure. A contemporary line of investigation on the relationship between viewing and liking starts with the work of Shimojo et al. (2003), who demonstrated what they called the “gaze cascade effect,” in which the observer’s gaze is biased towards the to-be-chosen stimulus prior to the preference decision. In their experimental paradigm, two images of faces were presented side-by-side on the screen. Subjects were required to perform a two-alternative-forced-choice (2AFC) preference task, while freely viewing the displayed faces. The images were kept on the screen until the subject made a response. Analyzing the likelihood of gazing at the to-be-chosen item as opposed to the to-be-rejected item, Shimojo and colleagues found a gradual increase, significantly deviating from a likelihood of 0.5 (the neutral level) more than half a second before actually selecting the item, and approaching the maximum likelihood of one around the decision time. The researchers suggested that the gaze bias both expresses and influences the preference.

The basic phenomenon of gradually increasing gaze likelihood on the to-be-chosen item was replicated in a variety of forced-choice tasks with a range of stimuli, including human faces (Simion and Shimojo, 2006, 2007), naturalistic scenes including landscapes, people in daily life, architecture, and animals (Glaholt and Reingold, 2009, 2011; Schotter et al., 2010), abstract visual patterns (Morii and Sakagami, 2015), color cards (Zommara et al., 2018), and images of red-wine bottles and snacks (Onuma et al., 2017). Further evidence suggested that gaze fixation (i.e., effective exposure duration) is the critical factor, independent of gaze shifting (Nittono and Wada, 2009; Bird et al., 2012; Gunia and Murnighan, 2015; Saito et al., 2017). In other words, the gaze cascade effect does not depend on movements of the gaze. It does not matter how frequently the gaze moves, or whether the gaze moves; instead, the effect is due to the amount of time actually spent gazing at the objects.

Formally, the hypothesis of a relationship between gaze fixation and evaluative processing can be understood as an accumulator model of decision-making (Krajbich et al., 2010). Accumulator models have proved to be a powerful approach toward capturing the relationship between internal processes (measurable in neural activity) and choice behavior. Such models can explain both the choice made and the time taken to do so (Smith and Ratcliff, 2004). They are also compatible with neural data that suggest a growth of the strength of representations toward a decision threshold (Hanes and Schall, 1996; Churchland et al., 2008). Within this framework, the gaze as an overt manifestation of attention would increase the internal processing, and thereby promote the value of the object being looked at. Thus, when choosing the most attractive of a pair of faces, the subject’s gaze gradually develops a bias toward the face that will turn out to be the favorite.

To prove that the gaze bias not only expresses but also influences preference formation, Armel et al. (2008) manipulated the amount of time subjects were able to view the items in a 2AFC preference task. The researchers found a differential impact from the constrained viewing time depending on the type of stimuli. For choices between appetitive food items, items viewed longer were chosen more likely. In contrast, for aversive food items the trend reversed, items viewed longer being chosen less often. In follow-up research using functional magnetic resonance imaging (fMRI), Lim et al. (2011) again manipulated the viewing time in a binary choice task between appetitive food items, and found neural correlates in ventromedial prefrontal cortex and the ventral striatum, suggesting a fixation-dependent encoding of relative value (see also Sokol-Hessner et al., 2012).

In short, the literature on gaze bias with 2AFC tasks converges on a strong, causal relationship between viewing and preference formation. However, it is less clear to what extent this relationship is generalizable to other types of evaluative processing. Most conspicuously, the 2AFC tasks require a comparative evaluation with a direct competition between two items, such that subjects should select one item at the expense of the other. In this case, as suggested also by the fMRI data by Sokol-Hessner et al. (2012), it is likely that the gaze contributes to valuating the fixated item more than the non-fixated item. This would reflect a form of *relative* preference formation. It is unknown to what extent the gaze also contributes to valuating fixated items in the absence of direct competition, that is, in case the subjects have to express an *absolute* evaluation, only pertaining to the current item in a single-image display. To examine this issue, we propose it is necessary to use a single-image paradigm instead of a 2AFC task.

Another limitation of the 2AFC task, as raised by Van der Laan et al. (2015), is that it potentially confounds the processes of preference formation with the decision goal. Van der Laan and colleagues illustrated this point by showing that the relationship between gaze fixation and choice was not uniquely related to preference formation. Indeed, even when subjects were required to indicate their least-preferred item, they gazed longer at the chosen item. Here, longer gazing did not lead to preference formation, but operated as a function of the decision goal, preparing the choice to be made. Especially in a 2AFC task, the gaze may contribute to preparing the spatial movement, required when one item should be picked from a display in which there are multiple items, regardless of whether this is based on liking (i.e., a precursor to spatial selection; see Gerbella et al., 2017). Then, any relationship between gaze and choice in a 2AFC preference task could reflect the preference formation, the spatial precursor function, or both. In order to disentangle the preference formation from spatial choice, we decided to use a single-image paradigm instead of a 2AFC task.

Effectively, our primary research question was whether the purported link between gaze and liking applies to absolute evaluative processing, with single images, under conditions that require no spatial selection on the screen. The corollary of the fMRI findings by Lim et al. (2011) is that the relationship may not necessarily hold for absolute evaluative processing. Indeed, we hypothesized there is no intrinsic connection between orienting and increased evaluation for several reasons.

Classic work by Posner (1980) has established a distinction between *overt* attention, locked to the gaze, and *covert* attention, an internal selection mechanism, usually joint with overt attention, but in fact separable from the focus point of the gaze. In other words, the focus of internal processing does not necessarily match with the focus of overt attention. Moreover, Treisman and Gelade (1980), in their “feature-integration theory,” launched the concept of attention as an operational mechanism with a binding function. Attention, in this view, serves to integrate information with respect to selected objects. Attention, then, should not necessarily increase the value of the attended object. Intriguingly, a study by Corneille et al. (2009) sheds light on the information-integrative function of attention with food images presented, not in a 2AFC task, but in an affective priming paradigm with only one food image per display. Here, it was found that the valence of products (peppermint brands) increased or decreased as a function of positive or negative associated information. The gaze did not necessarily lead to more liking, but contributed to the evaluative processing by integrating extra information.

To examine our hypothesis that viewing would not necessarily lead to increased liking in absolute evaluative processing, we designed a study with four types of evaluation. We varied the type of exposure. Subjects were either free to view the images at their own pace, or forced to view the images for a computer-generated duration. We also varied the nature of the type of evaluation. Subjects were either asked to give a rating (i.e., non-exclusive; with no restriction on the number of items that can receive a positive evaluation), or to pick a pre-determined number of items (exclusive; with a cap on the number of choices).

Finally, in the present study we opted to use naturalistic food images for evaluative processing for both practical and theoretical reasons. Our lab is engaged in a comprehensive research project on the evaluative processing of food as an important domain of health and consumer behavior; additionally, we were able to use a well-established database of food images (Blechert et al., 2014). Theoretically, we noted the advantage of using food stimuli as stimuli for complex evaluative processing (Suzuki et al., 2017), which may involve integrating non-visual information (e.g., memory recall relating to beliefs about flavor and nutritive attributes). Thus, we reasoned that food images provided us with a suitable opportunity to create a strong test of the relation between viewing and liking.

## Materials and Methods

### Participants

All 78 subjects were students from Kyushu University (37 females and 41 males; *M*_years_ = 21.7 ± 3.0). The subjects had normal or corrected-to-normal vision. No subject reported a diagnosis of any eating disorder, sleep deprivation or past or present neuropsychological disorder. The study was conducted in accordance with the ethical principles of Kyushu University and the Declaration of Helsinki. Written informed consent was obtained from each subject. All students received either course credit or a monetary reward of 1,000 yen for their participation.

### Stimuli

Stimuli consisted of a set of 320 naturalistic food images with a resolution of 600 × 450 pixels (96 dpi, standard Red Green Blue (sRGB) color format). The set of images was drawn from a food-pictures database for experimental research (Blechert et al., 2014) that comprised food images including: sweets (e.g., candies, ice cream, and chocolate), savory foods (e.g., pistachios, cheese straws), beverages (e.g., orange juice, iced café latte), processed foods (e.g., French fries, potato chips), and whole foods (e.g., vegetables, fruits). The food-pictures database includes extensive data regarding objective and subjective characteristics of the images. In selecting the 320 images from the set of 568 we endeavored to include images that would look most familiar to an Asian audience (mostly Japanese, but also Chinese, and other Asian students). This selection was made informally by discussion among lab members.

We created four sets of 80 pictures that showed no significant differences in any of the objective or subjective characteristics of the food-pictures database. For each participant, a different set of 80 pictures was used in each of the four evaluation tasks (see below for the definition of tasks). Thus, we ensured that subjects were never exposed to the same food image twice. The allocation of picture sets to tasks was counterbalanced; the order of food pictures was randomized within each task; and the order of the tasks was counterbalanced across subjects. Images were presented as a single stimulus on a black background.

### Apparatus

The visual stimulus was presented on a 23.8-inch full high definition (FHD) flat-panel-monitor, with a display resolution of 1920 × 1080 pixels. The subjects were seated approximately 65 cm from the monitor. To minimize head movement a chin-rest with a forehead-support was used. A keyboard set up was used to record the subjects’ responses.

In the initial phase of the experiment, for 34 subjects, we recorded only manual (keyboard) responses due to logistic limitations. However, to establish the amount of time actually spent gazing at each stimulus, we reckoned it was necessary to add an eye-tracking device to the experimental set-up. For the next 44 subjects, we were able to record manual (keyboard) responses as well as gaze position using Eye Tribe, an eye-tracking device at 60 Hz sampling rate (The Eye Tribe Aps, Denmark); a system with sufficient reliability for present purposes (Ooms et al., 2015; Zommara et al., 2018). Thus, the present data set includes a total of 78 subjects: 34 subjects with only manual responses, and 44 subjects with eye-tracking data in addition to the manual responses.

Before the start of a session with eye tracking, the subject was asked to follow a dot on the screen for a 12-point calibration. After the calibration, the gaze coordinates were calculated through Eye Tribe with an average accuracy of less than 0.5° visual angle on a 24-inch display. To prevent heat buildup a small universal serial bus (USB) fan was used. All events and recordings were controlled through code written in Psychopy (version 1.84.2); for reference, see Peirce (2007, 2009).

To compute actual viewing time (time with eye position on the displayed naturalistic food image) raw data were filtered. First, eye positions beyond the presentation area were removed. Second, detected and recorded eye blinks were also removed from the amount of actual viewing time if they lasted longer than 50 ms. Finally, the obtained data were plotted using custom software, and statistical analyses were conducted.

### Design and Procedure

One experimental session consisted of four different evaluation tasks: two different types of decision (non-exclusive versus exclusive) performed under two different types of exposure duration (self-paced versus time-controlled). Each subject was asked to participate in each of the four evaluation tasks.

For all tasks, the subjects were asked to base their decisions on how much they liked the food images. The subjects were instructed to evaluate the appetitive appeal of the food images; this type of evaluative processing presumably involves a combination of individual food preferences and the esthetic properties of the images.

In the non-exclusive evaluation tasks, the subjects were asked to give each food image a rating from 1 (“*not like at all*”) to 5 (“*like very much*”). There was no limitation on the number of positive or high evaluations. In contrast, in the exclusive evaluation tasks, the subjects were asked to pick a maximum of 15 food images for a virtual “basket” – that is, a restricted set of items with a positive evaluation. The task instruction was modeled after serial-choice paradigms (e.g., Constantino and Daw, 2015), and had proved effective in eliciting reliable viewing-time differences as a function of choice during a pilot study in our lab on the evaluative processing of art images (Espinoza Torres, 2015, Unpublished).

In the self-paced evaluation tasks, the subjects could determine the length of time they viewed the images. In contrast, in the time-controlled evaluation tasks, the exposure duration was computer-generated.

The four evaluation tasks were completed in counterbalanced order. The entire session lasted approximately 50 min. Prior to each experiment an informed consent, established according to ethical principles, was obtained. Each subject completed a questionnaire designed to assess the reported health state (e.g., eyesight, amount of hours of sleep the night before the experiment, medication intake, and food allergies occurrence). Before each task, the experimenter provided the appropriate instructions. Additionally, for sessions with eye tracking, the eye tracker was calibrated and instructions to look at the displayed food image were given. In between tasks, there was a brief pause, while the experimenter set up the computer to run the next task.

#### Self-Paced Non-exclusive Evaluation

**FIGURE 1:**
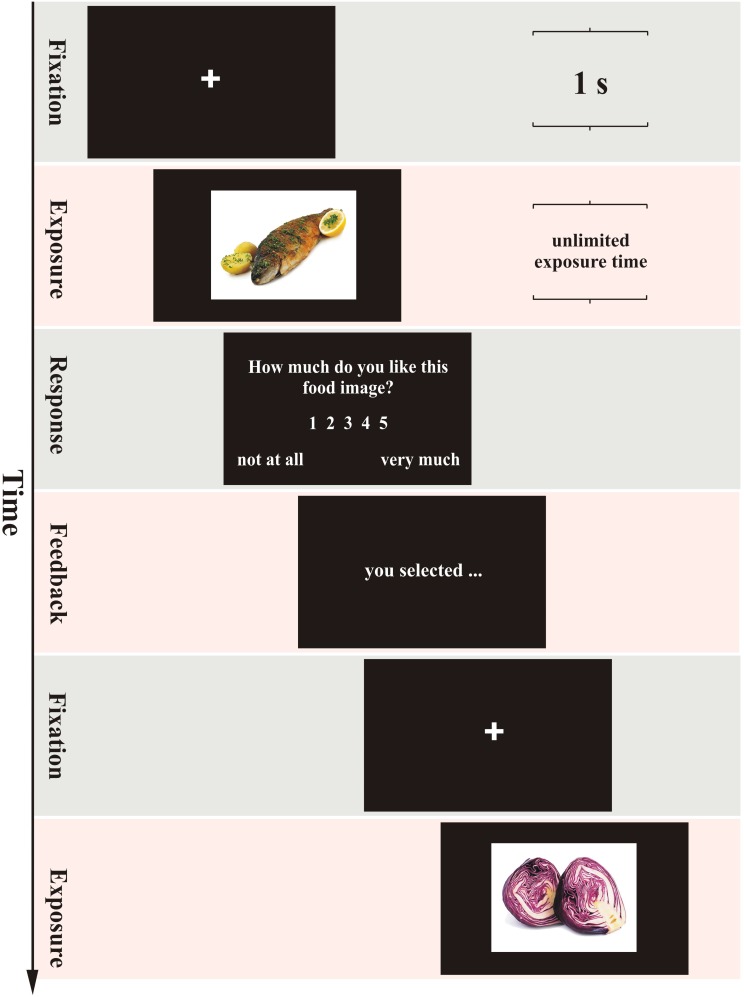
The trial structure in the self-paced non-exclusive evaluation. The structure was the same in the three other evaluation tasks except for the following critical differences. The duration of the exposure frame (second row) was either self-paced or time-controlled. In the self-paced evaluation tasks, the subject had to press the space bar to proceed to the response frame. In the time-controlled evaluation tasks, the response frame replaced the exposure frame automatically after a computer-generated duration. In the exclusive evaluation tasks, the response frame presented the question, “Would you like to add this image to your food basket?” and gave the options “Yes” or “No.” In the exclusive evaluation tasks, the feedback frame indicated the number of items added to the basket so far.

**Figure 1** presents a schematic overview of the sequence of events in the self-paced non-exclusive evaluation (SPN). In this task subjects were exposed to 80 naturalistic food images. Exposure time was defined as the length of time during which the food image was displayed on the screen. The self-paced task imposed no limit on the exposure time, so that subjects were able to look at each food image as long as they wished. The subjects indicated their readiness to move on to their evaluation by pressing the spacebar. After pressing the spacebar, the food image was removed, and the response screen appeared. On the response screen, the subjects were instructed to answer the question “How much do you like this food image?” by choosing a number from 1 (“*not like at all*”) to 5 (“*like very much*”). The question was shown in the absence of the food image. Subjects were required to respond by pressing a specific number, according to their rating, on the keyboard. There was no time limit for the response; that is, the response screen remained displayed until the subjects pressed a number between 1 and 5 on the keyboard. To confirm the recorded evaluation, a 1-sec feedback was displayed. Once a response was made, the image was replaced by the fixation cross.

#### Time-Controlled Non-exclusive Evaluation

This task was the same as the SPN, except for the exposure duration of the food images. Again, exposure time was defined as the length of time during which the food image was displayed on the screen. However, the food image on the stimulus screen was displayed for a pseudo-randomly chosen duration between 1 and 8 s, and was then automatically replaced by the response screen. Thus, in this task the subject had no control over the exposure time. We used only integer values for the exposure duration, ensuring each value between 1 and 8 was used 10 times over the course of 80 trials.

#### Self-Paced Exclusive Evaluation (SPE)

The question in the SPE was “Would you like to add this food image to your basket?” To make a choice, subjects were required to answer by pressing one of two specific keys: N (for “No”) or Y (for “Yes”). The aim of this task was to impose a limitation on the number of items that could receive a positive evaluation. For this purpose, the experimenter instructed all subjects to select a maximum of 15 food images. To confirm the choice and update the number of selected food images, a 1-sec feedback was presented after every decision (e.g., for the first selected image: “*1 out of 15*”). The task ended when the subject had selected 15 images, or when the subject had been presented with all 80 images. A pilot study in our lab, using a similar evaluation task with art images (Espinoza Torres, 2015, Unpublished), had shown that more than 90% of subjects picked the maximum of 15 items and viewed at least 30 items, offering a larger sample of rejected than included items.

In all other respects, this task was the same as the SPN. Exposure time was defined as the length of time during which the food image was displayed on the screen; the food image was removed at the time when the subject pressed the spacebar to move on to the response screen.

#### Time-Controlled Exclusive Evaluation (TCE)

In this task, the required choice was the same as in the SPE. With respect to the timing of the exposure duration, the task was the same as the time-controlled non-exclusive evaluation (TCN).

## Results

### Overall

Preliminary analyses showed that the data pattern for the 34 subjects with only manual responses replicated the data pattern for the 44 subjects with manual responses plus eye tracking (see **Supplementary Data Sheet S1**). For this reason, the two data sets were merged in the present analyses of exposure time. All subjects completed 80 trials in both the SPN and the TCN. All subjects completed the SPE (number of items picked for the basket: *M* = 14.63, SD = 1.30; number of trials viewed: *M* = 50.76; *SD* = 20.26). The TCE had to be aborted for three subjects due to complications with eye tracking. The remaining 75 subjects completed the task (number of items picked for the basket: *M* = 14.89, SD = 0.51; number of trials viewed: *M* = 45.03; SD = 16.66).

### Self-Paced Non-exclusive Evaluation (SPN)

#### SPN Exposure Time

**FIGURE 2:**
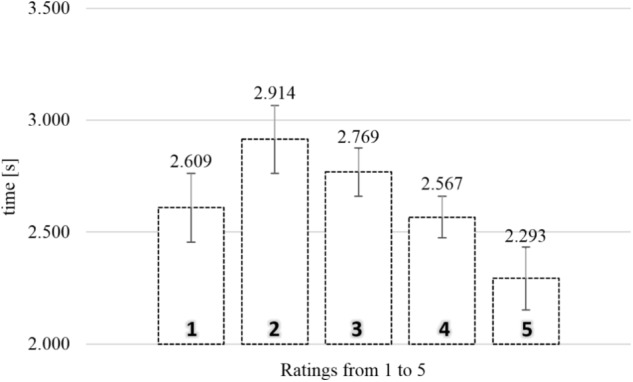
Average exposure time of food images rated from 1 (“not like at all”) to 5 (“like very much”) in the self-paced non-exclusive evaluation (*N* = 78). Error bars reflect the 95% confidence interval around the mean.

**Figure 2** shows the average exposure times of food images as a function of rating in the SPN. To analyze the relationship between rating and exposure time, a one-way repeated measures analysis of variance (ANOVA) was conducted with five levels of rating (from 1, “not like at all,” to 5, “like very much”), using the average exposure times for each subject for each level of rating as dependent measure. There was a significant relationship between rating and exposure time, *F*(4,308) = 9.946, MSE = 0.428, ${\text{η}}_{\text{p}}^{2}$ = 0.114, and *p* < 0.0001.

To gain further insights in the observed relationship between rating and exposure time, we analyzed the polynomial contrasts. The linear contrast, *F*(1,77) = 12.032, MSE = 0.623, ${\text{η}}_{\text{p}}^{2}$ = 0.135, and *p* < 0.005, and the quadratic contrast, *F*(1,77) = 14.003, MSE = 0.587, ${\text{η}}_{\text{p}}^{2}$ = 0.154, and *p* < 0.0001, were significant. The cubic contrast, *F*(1,77) = 3.504, MSE = 0.318, and *p* = 0.065, and the order four contrast, *F*(1,77) = 1.035, MSE = 0.183, and *p* = 0.312, were not significant. The polynomial contrasts indicated a trend such that higher ratings were associated with shorter exposure durations, compounded by an inverted *U*-shape tendency. Especially ratings two (2.91 s) and three (2.77 s) were associated with long exposure durations.

#### SPN Actual Viewing Time

**FIGURE 3:**
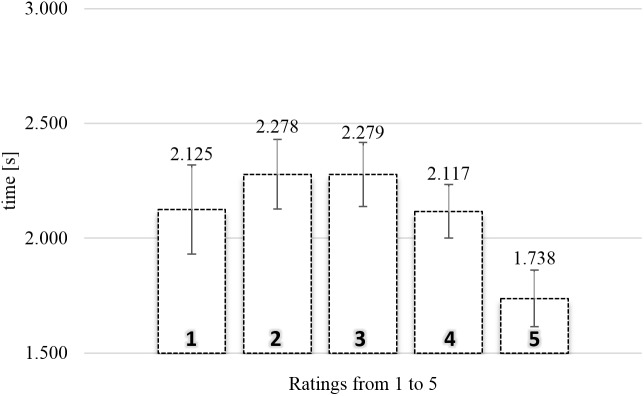
Average actual viewing time of naturalistic food images rated from 1 (“not like at all”) to 5 (“like very much”) in the self-paced non-exclusive evaluation (*N* = 44). Error bars reflect the 95% confidence interval around the mean.

**Figure 3** shows the average actual viewing times of food images as a function of rating in the SPN. A one-way repeated measures Analysis of variance (ANOVA) was conducted with five levels of rating (from 1, “not like at all,” to 5, “like very much”), using the average actual viewing times for each subject for each level of rating as dependent measure. There was a significant relationship between rating and actual viewing time, *F*(4,172) = 7.368, MSE = 0.291, ${\text{η}}_{\text{p}}^{2}$ = 0.146, and *p* < 0.0001.

To gain further insights in the observed relationship between rating and actual viewing time, we analyzed the polynomial contrasts. The linear contrast, *F*(1,43) = 11.126, MSE = 0.346, ${\text{η}}_{\text{p}}^{2}$ = 0.206, and *p* < 0.005, and the quadratic contrast, *F*(1,43) = 9.179, MSE = 0.514, ${\text{η}}_{\text{p}}^{2}$ = 0.176, and *p* < 0.005, were significant. The cubic contrast, *F* < 1, and the order four contrast, *F* < 1, were not significant. Again, the polynomial contrasts showed a trend such that higher ratings were associated with shorter actual viewing times, compounded by an inverted *U*-shape tendency. Especially ratings two (2.28 s) and three (also 2.28 s) were associated with long gaze durations.

### Time-Controlled Non-exclusive Evaluation (TCN)

#### TCN Exposure Time

**FIGURE 4:**
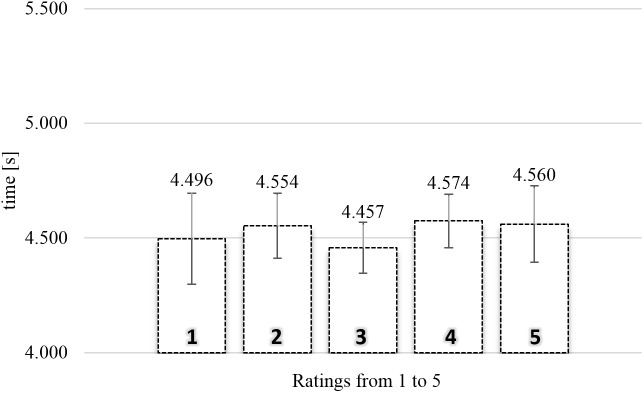
Average exposure time of food images rated from 1 (“not like at all”) to 5 (“like very much”) in the time-controlled non-exclusive evaluation (*N* = 78). Error bars reflect the 95% confidence interval around the mean.

**Figure 4** shows the average exposure times of food images as a function of rating in the TCN. A one-way repeated measures ANOVA with five levels of rating (from 1, “not like at all,” to 5, “like very much”), using the average exposure times for each subject for each level of rating as dependent measure, indicated there was no significant relationship between rating and exposure time, *F* < 1.

#### TCN Actual Viewing Time

**FIGURE 5:**
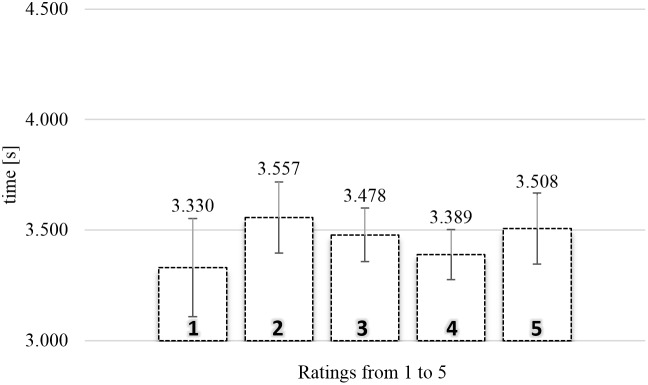
Average actual viewing time of naturalistic food images rated from 1 (“not like at all”) to 5 (“like very much”) in the time-controlled non-exclusive evaluation (*N* = 44). Error bars reflect the 95% confidence interval around the mean.

**Figure 5** shows the average actual viewing times of food images as a function of rating in the TCN. A one-way repeated measures ANOVA with five levels of rating (from 1, “not like at all,” to 5, “like very much”), using the average actual viewing times for each subject for each level of rating as dependent measure, confirmed there was no significant relationship between rating and gaze duration, *F*(4,172) = 1.052, MSE = 0.350, and *p* = 0.382.

### Self-Paced Exclusive Evaluation (SPE)

#### SPE Exposure Time

**FIGURE 6:**
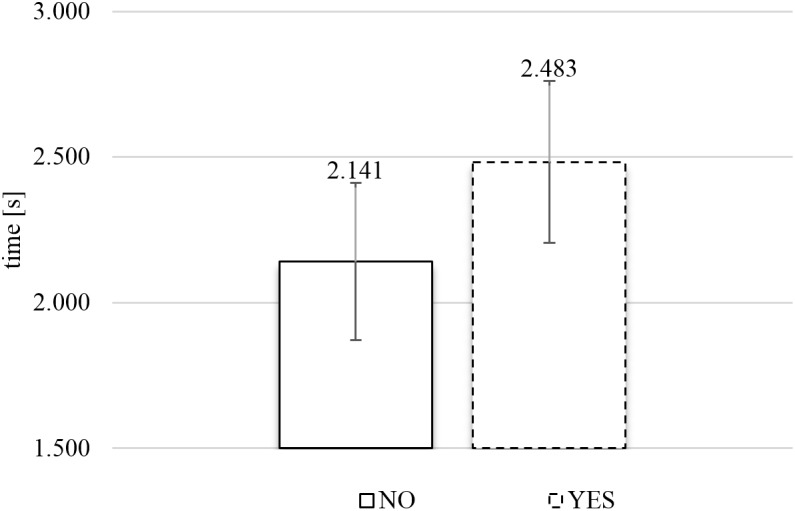
Average exposure time of rejected (NO) and included (YES) naturalistic food images in the self-paced exclusive evaluation (*N* = 78). Error bars reflect the 95% confidence interval around the mean.

**Figure 6** presents the average exposure times of food images as a function of response category (rejection or inclusion) in the SPE. A two-tailed paired *t*-test, comparing the average exposure times for each subject for YES versus NO responses, established there was a significant relationship between response category and exposure time, *t*(77) = 4.543, *p* < 0.0001, and Cohen’s *d* = 0.281. The data with the SPE showed that subjects spent significantly more time viewing images that they included (*M*_YES_ = 2.48 s) than images that they rejected (*M*_NO_ = 2.14 s).

#### SPE Actual Viewing Time

**FIGURE 7:**
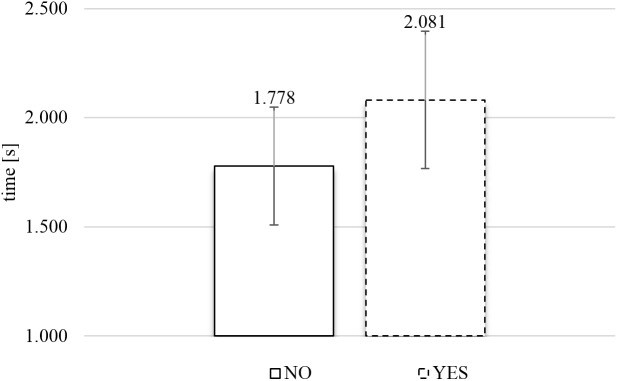
Average actual viewing time of rejected (NO) and included (YES) images in the self-paced exclusive evaluation (*N* = 44). Error bars reflect the 95% confidence interval around the mean.

**Figure 7** presents the average actual viewing times of food images as a function of response category (rejection or inclusion) in the SPE. A two-tailed paired *t*-test, comparing the average gaze durations for each subject for YES versus NO responses, confirmed there was a significant relationship between response category and actual viewing time, *t*(43) = 4.049, *p* < 0.0005, and Cohen’s *d* = 0.309. The data with the SPE showed that subjects spent significantly more time actually gazing at images that they included (*M*_Y ES_ = 2.08 s) than at images that they rejected (*M*_NO_ = 1.78 s).

### Time-Controlled Exclusive Evaluation (TCE)

#### TCE Exposure Time

**FIGURE 8:**
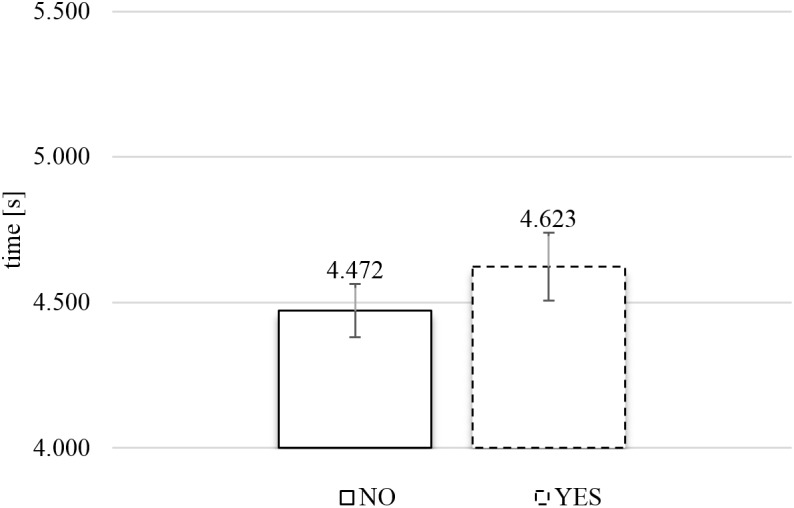
Average exposure time of rejected (NO) and included (YES) naturalistic food images in the time-controlled exclusive evaluation (*N* = 75). Error bars reflect the 95% confidence interval around the mean.

**Figure 8** presents the average exposure times of food images as a function of response category (rejection or inclusion) in the TCE. A two-tailed paired *t*-test, comparing the average exposure times for each subject for YES versus NO responses, produced no significant relationship between response category and exposure time, *t*(74) = 1.728, *p* = 0.088.

#### TCE Actual Viewing Time

**FIGURE 9:**
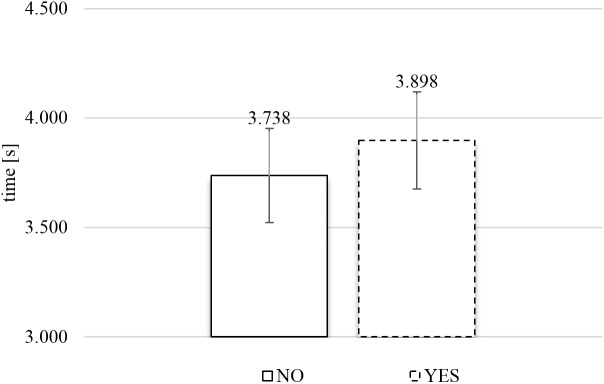
Average actual viewing time of rejected (NO) and included (YES) images in the time-controlled exclusive evaluation (*N* = 41). Error bars reflect the 95% confidence interval around the mean.

**Figure 9** presents the average actual viewing times of food images as a function of response category (rejection or inclusion) in the TCE. A two-tailed paired *t*-test, comparing the average actual viewing times for each subject for YES versus NO responses, confirmed that there was no significant relationship between response category and gaze duration, *t*(40) = 1.525, *p* = 0.135.

## Discussion

The goal of our research was to gain insight into the role of viewing in the evaluative processing of food images. In our experimental paradigm, we varied both the type of exposure (self-paced versus time-controlled) and the evaluative task (non-exclusive versus exclusive). A common notion in the literature on evaluative decision-making is that the longer a participant looks at an item, the more likely (s)he is to develop a preference for it. However, this proposal has been derived on the basis of 2AFC evaluation tasks in which subjects were required to indicate a preference for one of two items presented simultaneously. This literature, then, converges on the proposal that viewing leads to *relative* preference formation (Lim et al., 2011). Here, we tested the relationship between viewing and evaluative processing in tasks that did not involve an immediate comparison between multiple items on the screen. Using evaluation tasks with only a single image on the display, we aimed to examine the role of viewing in *absolute* preference formation.

With our single-image paradigm, we found that the relationship between viewing and evaluative processing depended on the type of task. In the SPE, we obtained a trend similar to the 2AFC findings, with longer viewing leading to a higher likelihood of a positive evaluation (i.e., inclusion in a virtual basket). In the time-controlled tasks, both with non-exclusive and exclusive evaluation, there was no significant relationship between the viewing duration and the evaluation. Moreover, in the SPN, when subjects were asked to give a rating from 1 to 5 (with no limitation on the number of positive evaluations), we obtained a significant relationship in the opposite direction, such that longer viewing durations were associated with lower ratings. The pattern of results was the same for viewing times measured in terms of exposure duration (i.e., the duration of stimulus presentation on the screen) and in terms of actual gaze duration (i.e., the amount of time the subject effectively gazed at the stimulus on the screen).

The present evidence indicates that viewing does not intrinsically lead to increased liking when evaluating single food images. Given that the controlled exposure durations did not influence the evaluation, and that the purported relationship between viewing and liking even went in the opposite direction in the SPN, we can firmly conclude that there is no mandatory connection between gaze and increase in absolute value. Our data, then, taken together with the robust findings from 2AFC paradigms, point to a critical distinction between absolute and relative evaluative processing. Previous research had established that the comparative mode, in which multiple items are considered simultaneously, crucially relies on the gaze for fixation-dependent accumulation of relative value. While making direct comparisons, the gaze may express and influence the competition or the selection among multiple items, as according to the proposal by Lim et al. (2011). The corollary of this notion is that viewing may not necessarily lead to more liking when the gaze does not need to select one from two or more simultaneously presented items. Our data show that, without the direct visual competition, the relationship between gaze and liking appears more complex. In the present study, both the self-determination of viewing time and the type of evaluation proved to be critical factors.

### Self-Determination of Viewing Time

With the comparison of the self-paced versus time-controlled tasks, we aimed to examine the role of self-determination in the relationship between viewing and evaluation. Importantly, by artificially imposing pseudo-random exposure durations between 1 and 8 s, our time-controlled tasks did not introduce a deadline or any urgency in the decision-making. Rather, the imposed durations were likely to mismatch with the subjects’ natural time course of evaluative processing, with durations that could be either longer or shorter than the subjects would have chosen, if given the opportunity to determine the viewing time. In this sense, our present examination with respect to self-determination cannot easily be mapped onto conventional approaches of the speed-accuracy trade-off (Standage et al., 2015). Instead, our procedure is comparable to the manipulated exposure time in 2AFC tasks designed to test whether there is a causal relationship between viewing and relative evaluation (e.g., Armel et al., 2008).

Critically, we found that, without the possibility of self-determination, there was no connection between viewing time and evaluative processing. This was true in both the TCN (with ratings from 1 to 5) and the TCE (with the categorical rejection or inclusion in a virtual basket). Here, it is important to note that, in the time-controlled tasks, not only the exposure durations, but also the actual gaze durations on the images showed no connection to the evaluations. We suggest that, without control over the exposure duration, subjects may disengage their covert attention from the overt point of gaze fixation. Put differently, their mind may not necessarily be on the screen, even if their gaze is directed there. By this interpretation, the lack of a relationship between viewing and liking in the time-controlled tasks is due to a disconnection between visual processing and decision-making. For instance, if a displayed image remains on the monitor even though its value has already been formed, the internal processing may no longer have the same focus as overt attention.

### Rating Versus Limited Choice

In addition to the self-determination of viewing time, we found that the type of evaluation had a critical impact on the relationship between viewing and evaluative processing. In fact, we obtained opposite trends in the SPN versus the exclusive evaluation. In the SPE, the likelihood of inclusion increased with longer viewing times, representing effectively a conceptual replication of the well-known gaze cascade phenomenon in 2AFC evaluation tasks (Shimojo et al., 2003). However, in the SPN, we found that longer viewing times were associated with lower ratings.

Notably, in 2AFC evaluation tasks the choice for one option implies choosing against the other, that is, the 2AFC induces a zero-sum competition. In such a situation, the underlying mechanism likely involves a cumulative process, gradually building commitment to a choice. This kind of decision process may be regarded as confirmatory, in the preparation of action. The decision is set in motion only when sufficient evidence has been accumulated, exceeding an internal threshold, or passing a point of no return. The zero-sum competition is a key feature of this type of decision process; the underlying neural computations are expressed as likelihood ratios between mutually exclusive alternatives (Gold and Shadlen, 2001). The gaze, then, may be a suitable precursor to the decision, such that the longer the observer looks at one of the two options, the more likely (s)he is to choose that option. In this case, by default, the gaze may express and influence preference formation – exactly as suggested by Shimojo et al. (2003).

Here, we propose that similar computational mechanisms, and a similar link between gaze and preference formation, apply to the present exclusive evaluation task, in which subjects were required to decide categorically for each particular food image whether they wanted to include it, yes or no, in the limited virtual basket of maximally 15 images. This paradigm involves a type of serial decision-making in which alternatives are considered one by one instead of being compared simultaneously (e.g., Hayden et al., 2011; Wikenheiser et al., 2013; Constantino and Daw, 2015). The question the subjects had to ask themselves for each item was whether to commit to the current option or wait for a better one. This class of problem, whether to stay or switch, occurs frequently in real-world settings, and is often analyzed in economics in terms of opportunity costs. We suggest that any categorical decision to include the current option may depend on a cumulative evaluation process that is structurally similar to the relative evaluation in 2AFC, with the difference that the comparison is not between two items, but between the current item and an internal threshold for acceptance. Future research should be able to characterize the underlying mechanisms (particularly through fMRI research akin to the work by Lim et al., 2011).

In contrast, in the non-exclusive evaluation, there was no limitation on the number of positive evaluations. The rating given to any image should have no implications for the other ratings. Subjects were always free to give any image the maximum score of five. In this situation, there was no critical need of confirmation, and no sense of a crucial point of no return. With no reason to hesitate, when an image looked good at first glance, the subject might as well give it a five right away. Our data show that particularly the highest rating of five was associated with brief viewing (less than 2 s actual viewing time) in the SPN. Such rapid evaluative processing may have been enabled by the nature of the decision, which implied no cost. There was no risk or potential loss of opportunity associated with giving a positive evaluation.

The findings with the present evaluation tasks also raise several important questions with respect to the underlying mechanisms that must be addressed in future research. Particularly, in the present paradigm we did not control for the initial preference with respect to the food images. In this sense, we cannot dissociate the evaluative processing in terms of *de novo* preference formation (i.e., a newly developing increment of value) versus preference formation based on retrieving prior knowledge (i.e., an allocation of value by comparing with previously stored information). To some extent, one might argue that *de novo* preference formation can only occur for categories of stimuli that are entirely unfamiliar to subjects (unlike the food-image database employed in the present study). However, it should be possible to investigate to what extent the evaluative processing relies on memory and recognition processes by systemically varying the familiarity and reinforcement learning with different types of stimuli.

Another concern with the present paradigm is that we instructed subjects to evaluate the attractiveness of the food images. On the response screen we labeled the to-be-evaluated stimulus explicitly as “food image.” Consequently, we cannot dissociate to what extent the evaluative processing was influenced by intrinsic food-related characteristics versus esthetic properties of the image. Although all subjects engaged in evaluative processing, it is possible that some subjects weighed primarily the esthetic dimension of the images, while other subjects may have based their evaluations more strongly on intrinsic properties of the food items. Food images likely represent a type of stimulus with distinctive features as compared to other visual images. The attractiveness of food images may inherently be a complex property, determined by visual as well as a range of non-visual characteristics. It will require an extensive research program to fully understand the complexity of the attractiveness of food images. The present study offers a suitable paradigm for such a research program. As notes for departure, we observe that the self-determination of viewing time and the type of decision task critically influence the relationship between viewing and absolute evaluation of food images.

## Ethics Statement

This study was carried out in accordance with the Declaration of Helsinki and the ethics guidelines of the Graduate School of Systems Life Sciences, Kyushu University. All subjects gave written informed consent (regarding the purpose of the research, expected duration, procedures, and confidentiality).

## Author Contributions

All authors contributed to the design of the study. AW conducted the data collection for the study, analyzed the behavior and eye-tracking data, and prepared all figures. AW and KO programmed the experiments. AW and JL wrote the manuscript. All authors reviewed and approved the manuscript.

## Conflict of Interest Statement

The authors declare that the research was conducted in the absence of any commercial or financial relationships that could be construed as a potential conflict of interest.

## Supplementary Material

DATA SHEET S1 Complete data set for the present study, organized in 8 Data Sheets: SPN_exp, Self-Paced Non-Exclusive Evaluation exposure duration; SPN_gaz, Self-Paced Non-Exclusive Evaluation actual viewing time; TCN_exp, Time-Controlled Non-Exclusive Evaluation exposure duration; TCN_gaz, Time-Controlled Non-Exclusive Evaluation actual viewing time; SPE_exp, Self-Paced Exclusive Evaluation exposure duration; SPE_gaz, Self-Paced Exclusive Evaluation actual viewing time; TCE_exp, Time-Controlled Exclusive Evaluation exposure duration; TCE_gaz, Time-Controlled Exclusive Evaluation actual viewing time.
